# Associations between good days per month and migraine-related disease impact in the real-world: a post hoc analysis from the REVIEW study of eptinezumab in chronic migraine

**DOI:** 10.1186/s10194-025-02227-5

**Published:** 2025-11-26

**Authors:** Fawad A. Khan, Steven P. Herzog, Ryan M. Smith, Dawn C. Buse, Seema Soni-Brahmbhatt, Susanne F. Awad, S. Wald Grossman, Divya Asher, Foram Patel, Charles Argoff

**Affiliations:** 1 The McCasland Family Comprehensive Headache Center, Ochsner Neurosciences Institute, Ochsner Health, New Orleans, LA USA; 2 The University of Queensland Medical School, Brisbane, Australia; 3 Texas Neurology, Dallas, TX USA; 4 St. Luke’s Health System, Meridian, ID USA; 5 Department of Neurology, Albert Einstein College of Medicine, Bronx, NY USA; 6 Lundbeck LLC, Deerfield, IL USA; 7 H. Lundbeck A/S, Copenhagen, Denmark; 8 Albany Medical Center, 47 New Scotland Ave, Albany, NY 12208 USA

**Keywords:** Chronic migraine, Eptinezumab, Real-world, Good days per month, Brain fog, Cognition, Patient satisfaction

## Abstract

**Background:**

Data from the real-world REVIEW (Real-world EVidence and Insights into Experiences With eptinezumab) study demonstrated that, after starting treatment with eptinezumab, individuals with chronic migraine (CM) experienced double the number of good days per month and high rates of satisfaction with eptinezumab. This post-hoc analysis explored the associations between self-reported changes in good days per month and other health-related outcome measures and the utility of good days per month as a patient-centric measure of the composite impact of migraine and migraine treatment on an individual.

**Methods:**

REVIEW was an observational, multi-site study designed to evaluate real-world experiences of patients treated with eptinezumab. A survey was administered to adults with CM who had completed ≥ 2 treatment cycles with eptinezumab. These post hoc analyses evaluated the impact of eptinezumab treatment on the ability to achieve a ≥ 50% and ≥ 75% increase in self-reported good days per month, and the association between these improvements and migraine symptoms, elements of daily living, overall well-being, and brain fog. Results are reported for the subgroups defined by the percentage change in self-reported good days per month after initiating eptinezumab treatment relative to before initiation (≥ 75% increase vs. < 75% and ≥ 50% increase vs. < 50%).

**Results:**

Following eptinezumab initiation, ≥ 75% and ≥ 50% increases in good days per month were reported by 59/92 (64%) and 70/92 (76%) evaluable participants, respectively. Participants with ≥ 75% increase in good days per month also reported a greater positive impact on multiple migraine symptom domains (68%–90%), as well as select aspects of daily living and overall well-being (73%–90%) compared with participants with < 75% increase (27%–61% and 18%–39%, respectively). Moderate to complete improvement in brain fog after eptinezumab was reported by 77% of participants in the ≥ 75% increase subgroup versus 42% in the < 75% subgroup. Results were consistent for the ≥ 50% increase versus < 50% subgroups.

**Conclusions:**

Following eptinezumab treatment, most participants reported substantial increases in good days per month, with those experiencing ≥ 75% improvement also reporting greater improvements across multiple migraine symptom domains, daily functioning, overall well-being, and brain fog. These real-world data underscore the importance of novel, patient-centered approaches to understand how preventive therapies can reduce multiple aspects of migraine burden.

**Trial registration:**

N/A.

**Supplementary Information:**

The online version contains supplementary material available at 10.1186/s10194-025-02227-5.

## Background

Migraine is a common and highly debilitating neurological disease presenting with diverse symptoms besides the core characteristic of headache pain, resulting in a wide potential range of negative impacts [1]. Gaining insight into the perspectives of people living with migraine on the relative importance of treatment benefits is fundamental to informing therapeutic innovation, optimizing clinical trial methodologies, and enhancing evidence-based clinical decision-making [2]. Current clinical benchmarks used to evaluate the effectiveness of migraine treatment emphasize counting the number of days with pain and symptoms (e.g., monthly migraine and/or headache days) to assess disease burden and reduction in these days to assess treatment response [3, 4]. Generally, these measures rely on accurate completion of headache diaries to provide the data necessary to calculate responder rates (e.g., reduction in the number of migraine days per month compared to baseline). In real-world studies, a headache diary may not always be used, and therefore proxy endpoints are needed to gauge effectiveness.

Individuals with migraine often experience disease-related burden in addition to headache pain that may include aspects such as reduced functionality, disability, and symptoms like cognitive impairment [5–8]. Neglecting the impact of these other symptoms can lead to failure in delivering patient-centered care [9]. Integrating the patient voice into migraine research is a key step in understanding goals for treating migraine, highlighting elements that are important to individuals, and tailoring treatment plans to reduce the migraine burden and improve patients’ lives in a meaningful way [1, 10, 11]. Patient-reported outcome measures (PROMs) provide a mechanism whereby researchers and healthcare providers can acquire a greater comprehension of patient perceptions of migraine symptoms, impact on daily life, and the effectiveness of treatment, allowing for greater shared decision making. However, while widely used within the clinical trial setting, the content and application of PROMs in real-world clinical care show considerable heterogeneity [12–14]. As such, alternative methods for gauging disease burden and tracking responses to treatment interventions are needed to improve outcomes in clinical practice settings [15].

In real-world practice, patients provide valuable clues that present opportunities to better understand their lived experiences and symptoms from chronic diseases, including migraine [16]. Traditionally, preventive trial endpoints have been focused on cardinal symptomatologies—pain, photophobia, nausea, and most bothersome symptoms—as well as the numerical attack experience per month, with “migraine days” or “headache days” serving as endpoints for response to interventions in both clinical practice and clinical trials. However, these endpoints may overlook subtle nuances related to patient perceptions of improvement and positive outcomes. If a counter-approach of assessing “good days” were used, alongside the usual metrics, it could provide a more patient-oriented perspective, offering a clearer, more holistic picture while addressing gaps in current practice. Moreover, for individuals with CM, who often experience frequent symptom days that may blend together, asking about good days may offer a more intuitive and meaningful way to recall and express the true burden of disease.

Eptinezumab is an intravenously administered monoclonal antibody targeting calcitonin gene-related peptide (CGRP), which is involved in migraine pain generation [17]. Data from multiple randomized clinical trials have confirmed the efficacy and safety of eptinezumab in various migraine populations, including individuals with chronic migraine (CM) and those in whom traditional oral prior preventive treatments have failed [18]. However, real-world data are important to fully understand the true impact of migraine preventive treatments in people living with migraine who are typically seen in a clinic—likely those who have tried other anti-CGRP therapies—to provide greater confidence to clinicians when choosing the most appropriate therapy for their patients.

The real-world REVIEW study evaluated patient-reported survey data obtained from individuals with CM and reported their experiences after initiating treatment with eptinezumab. Good days per month was introduced as a concept in REVIEW to capture a patient-centric, holistic, and positive-focused assessment of migraine burden, with the emphasis being on days when individuals can participate in their lives in a meaningful way. We have previously reported a two-fold increase in good days per month after starting eptinezumab treatment, and high rates of satisfaction with the effectiveness of treatment in improving measures such as symptoms, elements of daily living, overall well-being, and brain fog [19].

Current literature has established correlations between monthly migraine day (MMD) responder rates (≥ 50% or ≥ 75% reduction in MMDs) and improvements in many PROMs [20, 21]. This post-hoc analysis of REVIEW was conducted to explore whether an association also exists between percent improvements in good days per month and PROMs. In this analysis, we paralleled the ≥ 50% and ≥ 75% reduction in migraine days responder rates by examining ≥ 50% and ≥ 75% increases in self-reported good days per month. These thresholds were used as a proxy for measuring and benchmarking migraine improvement and to better understand the holistic impact of eptinezumab in reducing the burden of CM and to substantiate the use of this assessment.

## Methods

### Study design

Details of the observational, multi-site REVIEW (*R*eal-world *EV*idence and *I*nsights into *E*xperiences *W*ith eptinezumab) study have been previously published [19]. In brief, this was a real-world, observational study conducted at four tertiary headache centers in the United States and included a structured participant survey and a retrospective chart review, as well as interviews with healthcare providers. Herein, we report post hoc analyses based on data collected from the cross-sectional, post-eptinezumab participant survey, which was administered either electronically (one site) or at the time of infusion or office visit (three sites).

### Participants

Key eligibility criteria for REVIEW were age ≥ 18 years, a diagnosis of CM as shown in the chart review or adjudicated by the treating physician, and ≥ 6 months of exposure to eptinezumab (i.e., full completion of at least two eptinezumab infusion cycles). Individuals treated with eptinezumab in a clinical trial or who were currently enrolled in a migraine or headache clinical trial were excluded. All four study sites recruited eligible participants by selecting the first 25 participants, ordered by birth month, to obtain consent for survey administration.

#### Outcomes

This analysis evaluated the impact of eptinezumab treatment on the ability to achieve a ≥ 50% and ≥ 75% increase in self-reported good days per month, and the association between participant-reported increases in good days per month and changes in other outcomes related to migraine symptoms, daily living and overall well-being, and improvement in brain fog. The number of good days per month (from 1 to 31) before and after starting treatment with eptinezumab was specified by participants, with the exact interpretation of good days being left to each individual; no study specific definition was provided. The associations between increases in good days per month and changes in other outcomes were analyzed in subgroups defined by the increase in good days per month before versus after eptinezumab (≥ 75%, < 75%, ≥ 50%, or < 50%).

Participants rated their satisfaction with the impact of eptinezumab on migraine symptoms (5 domains) on a 5-point scale from strongly agree to strongly disagree. Satisfaction with the impact of eptinezumab on daily living and overall well-being was rated on a 5-point scale from much higher to much lower. To assess brain fog, participants were first asked “Have you experienced ‘brain fog’ (feeling confused, have difficulty learning or remembering, or have trouble speaking or reading) before starting on eptinezumab?”, with the response options of yes, no, or unsure. If the answer given to the question was yes, participants were then asked, “If yes, please rate to what extent your symptoms have improved since starting eptinezumab”, with the response options of completely, very much, moderately, slightly, or not at all.

### Statistical analysis

The analysis set included all participants who completed the survey, with results summarized descriptively. There was no imputation for missing data; only observed data were included. Continuous variables were summarized using the number of participants, mean and standard deviation, and median and range. Categorical variables were summarized using the absolute counts and percentages of participants who provided responses. Analyses were conducted using Microsoft Excel (version 10; Microsoft Corp., Redmond, WA), SAS software (version 9.4 or higher; SAS Institute, Cary, NC), R statistical software (version 4.3.1; R Core Team, Vienna, Austria), and Stata BE (version 17; StataCorp, College Station, TX).

## Results

### Study population

The REVIEW study included 94 participants, among whom 78 (83%) were female, the mean age was 49 years, 84 (89%) were White or Caucasian, and the mean time since CM diagnosis was 15 years; full demographic details have previously been published [19]. Overall, 93/94 participants answered the survey question relating to the presence of brain fog, among whom 74 (80%) reported experiencing this symptom prior to starting treatment with eptinezumab.

### Association of good days per month with other surveyed outcomes

In terms of good days per month, 92/94 REVIEW participants had data available for evaluation (two had missing data for the period either before or after starting eptinezumab treatment). We have previously reported that the mean number of good days per month was 8 before starting eptinezumab treatment, with this number increasing more than 2-fold to 18 after eptinezumab initiation [19]. Compared with the period before starting eptinezumab, the majority of participants (59/92 [64%]) had a ≥ 75% increase and 33/92 (36%) had a < 75% increase in good days per month after eptinezumab initiation. Similarly, 70/92 (76%) had a ≥ 50% increase and 22/92 (24%) had a < 50% increase in good days per month after eptinezumab initiation.

**Fig. 1:**
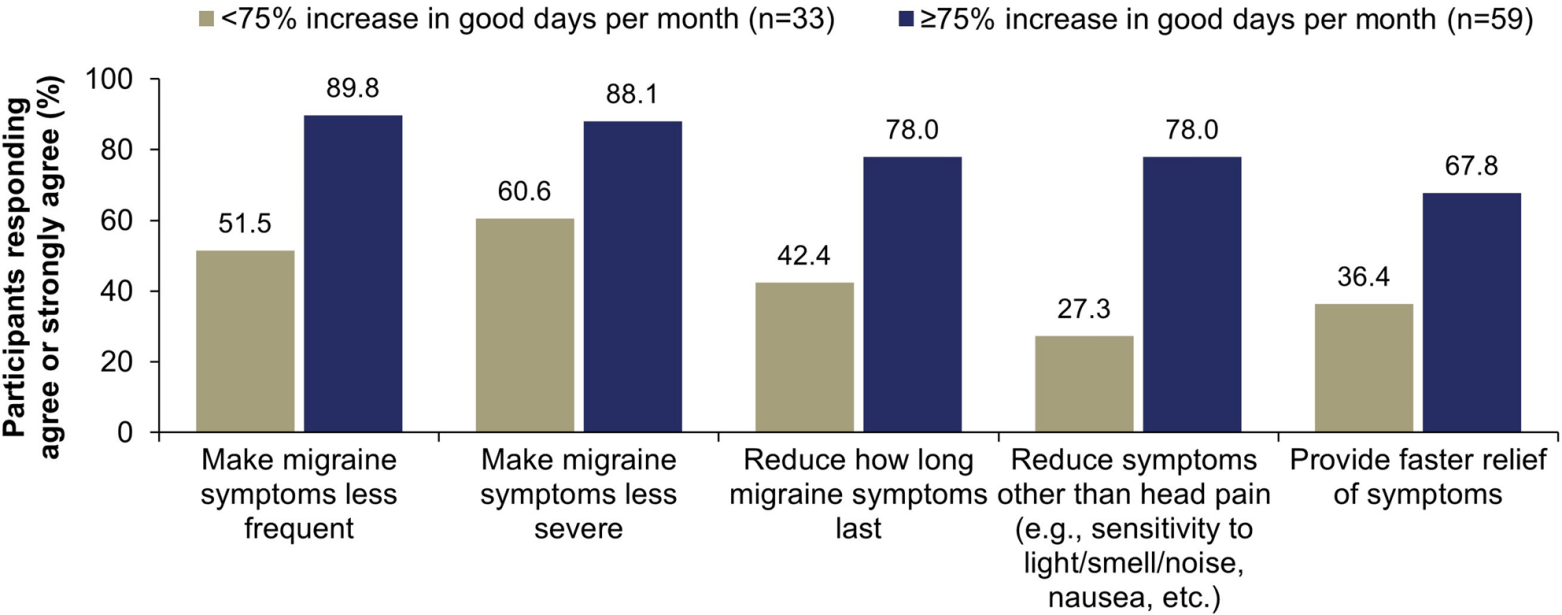
Association between < 75% or ≥ 75% increase in self-reported good days per month and satisfaction with the ability of eptinezumab to impact migraine symptoms. Participants were prompted: “Please rate how much you agree or disagree with the following statements by placing a checkmark ✓ in the column which most closely fits your opinion: I am satisfied with eptinezumab’s ability to…” Choices included: strongly agree, agree, neutral, disagree, and strongly disagree. Data shown here are for participants who chose strongly agree or agree

A greater percentage of participants with a ≥ 75% increase in good days per month reported satisfaction with the ability of eptinezumab to impact migraine symptoms (68%–90%) compared with participants with a < 75% increase (27%–61%) (Fig. 1). Similar results were observed when the ≥ 50% cut-off was used (Supplementary Fig. 1). For both the ≥ 75% and ≥ 50% cut-offs, the largest between-group differences were observed for the frequency of migraine symptoms and the reduction in symptoms other than head pain.

**Fig. 2:**
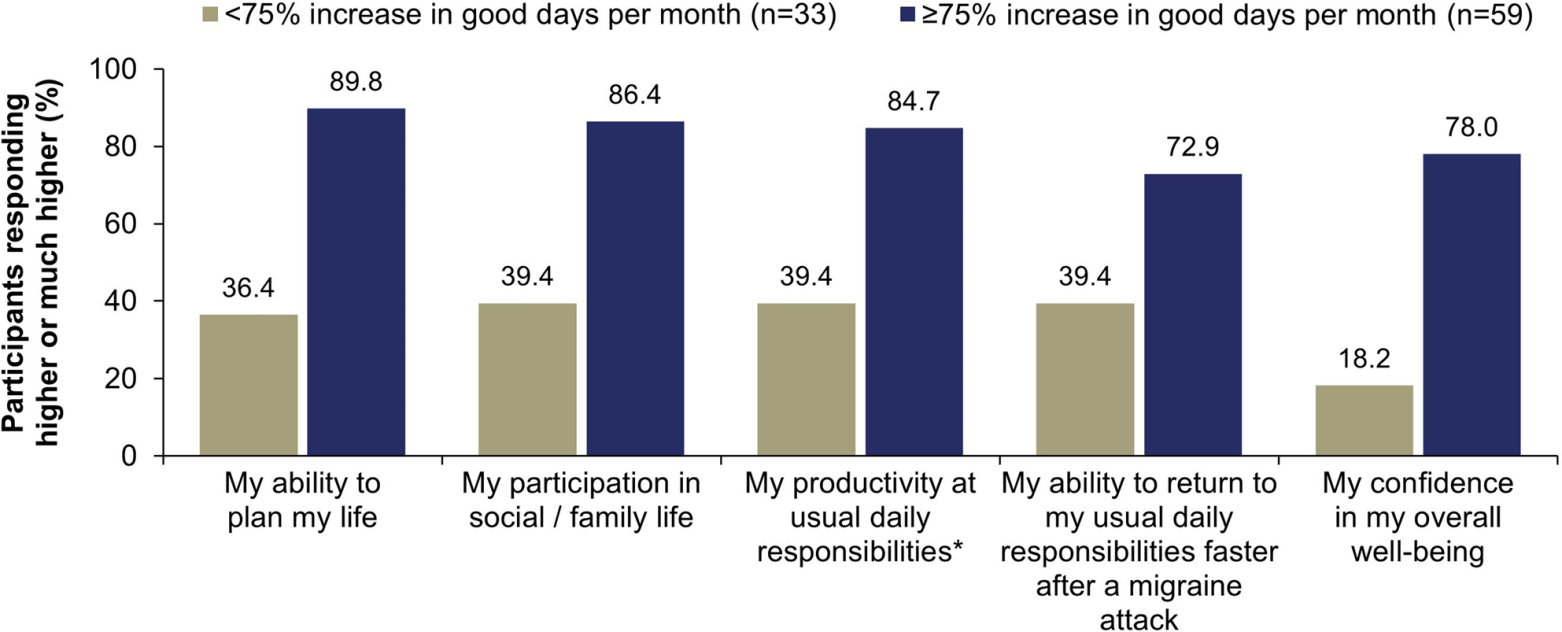
Association between < 75% or ≥ 75% increase in self-reported good days per month and satisfaction with daily living and overall well-being. *For example: school, work, taking care of children/family members. Participants were prompted: “Please rate the following statements on different aspects of your life (i.e., your feelings) by placing a checkmark ✓ in the column which most closely fits your opinion: After starting eptinezumab, my satisfaction with…” Choices included: much higher, higher, about the same, lower, and much lower. Data shown here are for participants who chose much higher or higher

A greater percentage of participants with a ≥ 75% increase in good days per month also reported a positive impact of eptinezumab on selected items that assessed daily living and overall well-being​ (73%–90%) compared with participants with a < 75% increase (18%–39%). For participants with a ≥ 75% increase in good days per month, 78% reported higher satisfaction with their overall well-being after starting eptinezumab treatment, compared with 18% of participants with a < 75% increase (Fig. 2). Similar trends were observed with the ≥ 50% cut-off (Supplementary Fig. 2).

**Fig. 3:**
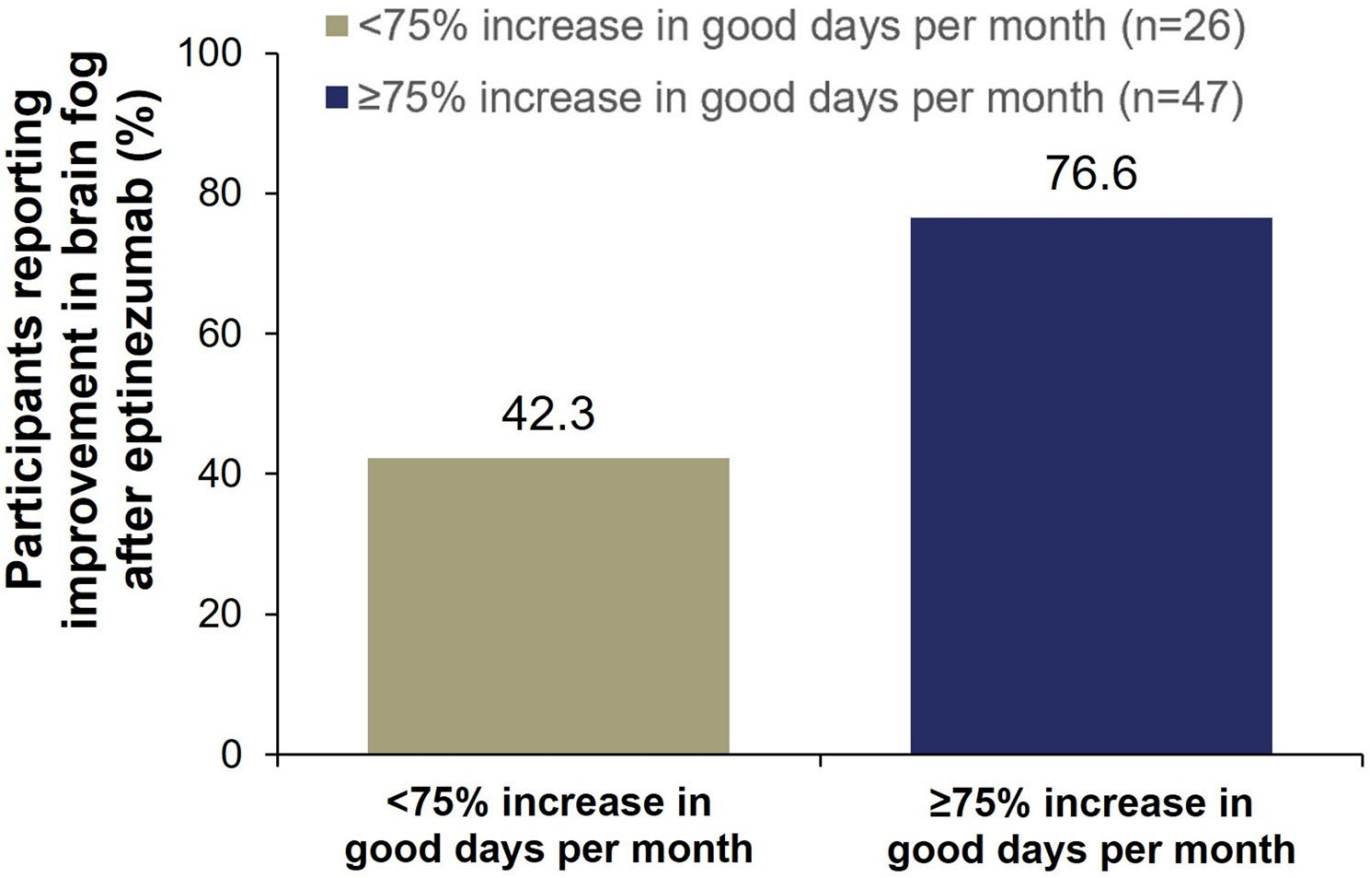
Association between < 75% or ≥ 75% increase in self-reported good days per month and moderate to complete improvement in brain fog after starting eptinezumab. Participant prompt: “Have you experienced ‘brain fog’ (feeling confused, have difficulty learning or remembering, or have trouble speaking or reading)? If yes, please rate to what extent your symptoms have improved since starting eptinezumab.” Moderate to complete improvement included the choices “completely,” “very much,” and “moderately”

The prevalence of brain fog before starting eptinezumab treatment was generally similar across subgroups (< 75% increase subgroup: *n* = 26/33 [79%]; ≥75% increase subgroup: *n* = 47/59 [80%]); however, in the ≥ 75% increase subgroup, 77% of participants reported moderate to complete improvement in brain fog after eptinezumab compared with 42% in the < 75% increase subgroup (Fig. 3). Similarly, 75% of participants in the ≥ 50% increase subgroup reported moderate to complete improvement in brain fog compared with 25% in the < 50% increase subgroup (Supplementary Fig. 3).

## Discussion

Our previous analysis of real-world data from the REVIEW study in individuals with CM found that initiation of eptinezumab treatment was associated with an increase in good days per month, along with greater satisfaction with the effect of eptinezumab on migraine symptoms, elements of daily living and overall well-being, and brain fog [19]. The results of this current post-hoc analysis build on this evidence, showing that almost two-thirds (64%) of REVIEW participants reported a ≥ 75% increase in good days per month following eptinezumab initiation and that more than three-quarters (76%) reported a ≥ 50% increase. Moreover, both ≥ 75% and ≥ 50% increases in good days per month were associated with greater improvements in migraine symptoms, as well as in aspects of daily living and overall well-being and in brain fog.

Traditional clinical trial endpoints include counting the number of days with migraine pain and associated symptoms to assess disease burden and treatment response. In theory, a reduction in MMDs suggests disease improvement, but it does not fully account for the patient’s lived experience, migraine-related disability, or overall sense of well-being. Furthermore, this approach assumes that non-symptomatic days are inherently “healthy,” without accounting for the variability in how patients feel on those days—some of which may still be impacted by mild symptoms, anticipatory anxiety, or reduced function. A more patient-centered approach is to ask the reverse question: how many good days per month does a patient experience? This shift provides insight into days where individuals with migraine may feel at their best—however they choose to individually define it—aligning more closely with their expectations and perceptions of their condition.

The number of participant-reported good days per month was used in the REVIEW study to obtain a more holistic view of the burden of migraine and treatment effectiveness, beyond simply the number and severity of migraine days. The interpretation of what constituted a “good day” was left to the discretion of participants and is likely to include subjective factors (e.g., cognition, daily functioning, and/or affects from common comorbidities) as well as migraine pain. Our findings suggest that tracking changes in good days per month may provide some insight into the broader impact of preventive therapies on a patient’s migraine experience, including potential changes in symptoms and quality of life, though further validation of this measure is needed.

We evaluated our data using both ≥ 75% and ≥ 50% cut-offs, which is similar to the methodology used when reporting responder rates in clinical trials [18, 22, 23]. While a ≥ 50% improvement threshold is comparable to the traditional standard threshold for treatment response, we found that the subgroup sizes were slightly unbalanced in the REVIEW participant population (*n* = 70 and *n* = 22 for ≥ 50% and < 50% increase in good days per month, respectively). The ≥ 75% improvement threshold provided a better balance of participant numbers (*n* = 59 and *n* = 33 for ≥ 75% and < 75% increase in good days per month, respectively). Furthermore, we assert that higher thresholds, such as ≥ 75% increases in good days per month, allow for the assessment on the ability to push the boundaries for preventive treatment efficacy by emphasizing meaningful disease improvements that newer migraine treatments should strive to achieve.

While tracking either good or bad days over time can illustrate trends and help guide treatment decisions, quantifying good days offers an additional advantage—it reflects the broader disease burden on overall well-being [5], which includes not only migraine-related symptoms but also symptoms of common comorbidities, including anxiety and/or depression. It also allows the patient to define what a “good day” is to them. In this regard, good days per month may serve as a more sensitive measure of disease impact, particularly for individuals with CM, who spend most days experiencing some degree of bothersome or debilitating symptoms. According to our post-hoc analysis, participants who reported ≥ 75% or ≥ 50% increases in good days per month also experienced greater improvements in migraine symptoms, daily living, and overall well-being compared to those who did not achieve these thresholds. One of the items that showed the largest between-group differences was the reduction of symptoms other than head pain, which implies that additional (non-headache) factors are a key part of the migraine burden, and that amelioration of these feed into the overall concept of a good day for participants. This is further supported by the differences between subgroups in satisfaction with daily life and overall well-being, with more than 70% of participants who reported ≥ 75% or ≥ 50% increases in good days per month also reporting confidence in their overall well-being, compared with fewer than 20% of participants who had < 75% or < 50% increases.

This analysis also evaluated the perceived impact of eptinezumab treatment on brain fog. Cognitive dysfunction has become recognized in recent years as an important and highly debilitating symptom associated with chronic conditions such as migraine [6, 24–27]. Because cognitive symptoms like brain fog can occur ictally and/or interictally [6, 27, 28], they negatively impact patients’ lives during both migraine days and non-headache days, further adding to the burden of migraine. As such, treatments that can address associated brain fog can meaningfully and holistically improve the migraine experience. Although a standard definition of brain fog does not exist, in this study we defined it as “feeling confused, have difficulty learning or remembering, or have trouble speaking or reading.” We found that three-quarters of participants in the ≥ 75% increase and ≥ 50% increase subgroups reported moderate to complete improvement in brain fog after eptinezumab compared with 42% in the < 75% increase subgroup and 25% in the < 50% increase subgroup, highlighting an important association in migraine, a disease known for its multidomain and widespread impacts on the brain. These findings support that brain fog is an important dimension of symptomatology and neuronal impairment in individuals with migraine, and of their experience of the impact of migraine. Not only is this symptom prevalent in migraine (~ 80% of individuals with CM in this study reported experiencing brain fog), but it is likely juxtaposed as a critical element when assessing the burden of migraine. Clinically and pragmatically, this underscores the importance of considering non-pain factors when evaluating the overall clinical impact of preventive migraine treatment.

The limitations of this study, including the exploratory nature of our analyses, the small survey population, the observational design, the specialized nature of the recruitment settings, and requiring a ≥ 6-month trial of eptinezumab, all restrict the inferences that can be drawn from these data and may allow for bias in participants (individuals who were not satisfied and who discontinued therapy after a single dose would not have been captured in this analysis). Moreover, the small sample size and descriptive design of the study limited the ability to draw statistically significant conclusions. In addition, the multiple-choice design of several of the survey questions restricted response options (e.g., response options for brain fog were only offered for staying the same or improving, but not for worsening), and given the survey was administered without further guidance, survey questions were subject to individual participant interpretation. More specifically, the interpretation of the term “good days,” which was left to each participant to determine individually, makes responses highly subjective, although it is important to note that all patient-reported outcomes in migraine are inherently subjective. Additionally, we recognize that 50% and 75% reductions in good days are not validated outcomes and do not correlate directly with MMD responder rates; however, these thresholds allowed us to explore associations between extent of response and other efficacy measures. Analyzing both “good days” and MMD outcomes in a single study could strengthen the interpretation of the “good days” measure; however, this was beyond the scope of the current analysis. The term “brain fog” is also subjective and was not defined in terms of severity, frequency, onset of symptom in relation to migraine attacks or migraine disease, or perceived cause of brain fog (e.g., migraine, another condition, migraine treatment, or other drug therapy). Response options for changes in brain fog were only offered for improvement and not worsening. Finally, all data in the survey were subject to participant recall, especially relating to the pre-eptinezumab period (which was ≥ 6 months prior to the survey completion), potentially introducing bias into the data.

## Conclusions

In conclusion, this post hoc analysis underscores the importance of incorporating patient-centered measures to more comprehensively capture the impact of migraine—addressing not only symptom burden but also overall perceived well-being. This is particularly meaningful in higher-frequency episodic migraine and CM, where the disease burden is high, and quality of life is significantly impacted. Such approaches allow for the assessment of therapeutic efficacy and benefits of preventive migraine treatments while tracking progress in mitigating the holistic burden of migraine beyond just headache pain. Assessing good days per month allows for the inclusion of a broad spectrum of positive outcomes that reflect what matters most to individuals with migraine. The response of ≥ 75% increase in good days per month was associated with corresponding improvements in a range of outcomes, including migraine symptoms, aspects of daily living, overall well-being, and brain fog highlighting the importance of pushing the boundaries on preventive treatment expectations beyond traditional thresholds. These findings invite further discussion and support ongoing scientific efforts to define and prioritize holistic treatment goals. Overall, these real-world data affirm that migraine is a multifaceted disease and that preventive therapies like eptinezumab can drive significant improvements across multiple dimensions of disease burden.

## Supplementary Information

Below is the link to the electronic supplementary material.

Supplementary Material 1

## Data Availability

The data sets generated and/or analyzed during the current study are not publicly available due to data license restrictions. However, summarized data are available from the corresponding author upon reasonable request.

## Abbreviations

CGRP: Calcitonin gene-related peptide
CM: Chronic migraine
MMD: Monthly migraine day
PROMs: Patient-reported outcome measures
