# Supplementary material for: Associations between good days per month and migraine-related disease impact in the real-world: a post hoc analysis from the REVIEW study of eptinezumab in chronic migraine
Source: J Headache Pain. 2025 Nov 26;26(1):291. doi: 10.1186/s10194-025-02227-5 (PMC12750955; doi:10.1186/s10194-025-02227-5)
Supplement: Supplementary file 1 — Supplementary Material 1 [file 10194_2025_2227_MOESM1_ESM.pdf]

Supplementary Material

Supplementary Figure 1. Association between <50% or ≥50% increase in self-reported good days per month and satisfaction with the ability of eptinezumab to impact migraine symptoms.

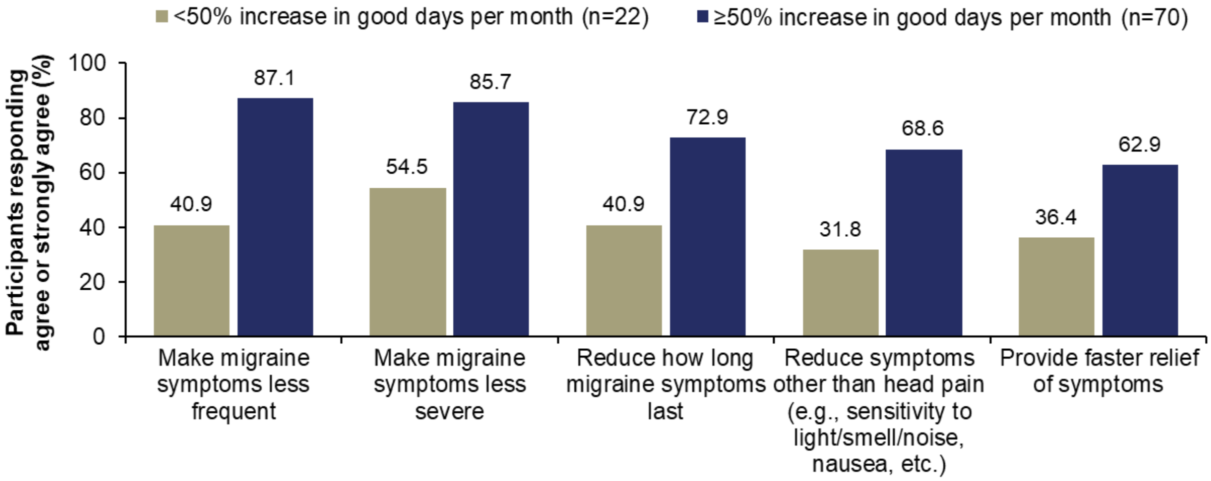

Participants were prompted: "Please rate how much you agree or disagree with the following statements by placing a checkmark ✓ in the column which most closely fits your opinion: I am satisfied with eptinezumab's ability to…" Choices included: strongly agree, agree, neutral, disagree, and strongly disagree. Data shown here are for participants who chose strongly agree or agree.

Supplementary Figure 2. Association between <50% or ≥50% increase in self-reported good days per month and satisfaction with daily living and overall well-being.

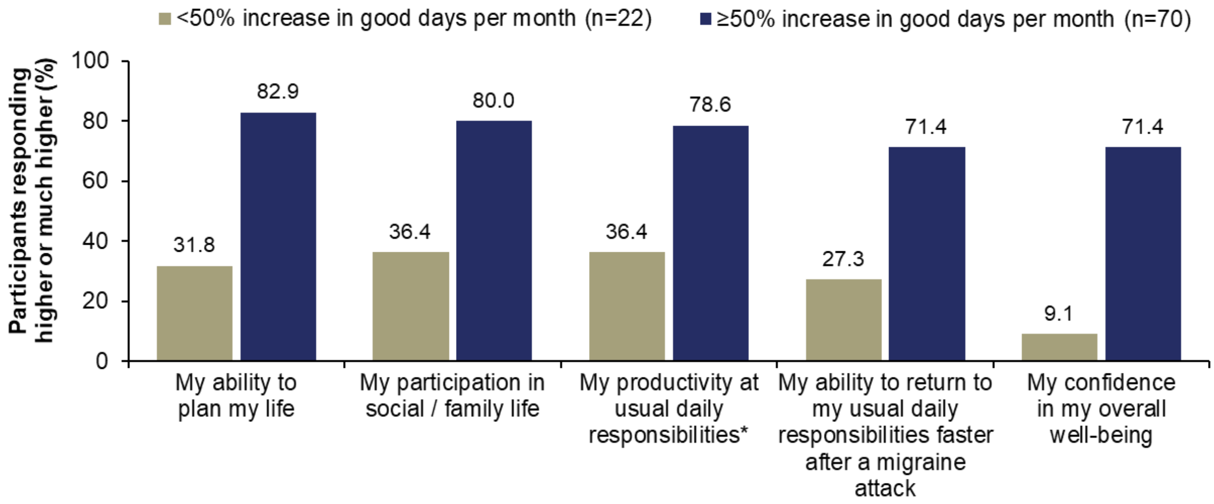

*For example: school, work, taking care of children/family members.

Participants were prompted: "Please rate the following statements on different aspects of your life (i.e., your feelings) by placing a checkmark ✓ in the column which most closely fits your opinion: After starting eptinezumab, my satisfaction with…" Choices included: much higher, higher, about the same, lower, and much lower. Data shown here are for participants who chose much higher or higher.

Supplementary Figure 3. Association between <50% or ≥50% increase in self-reported good days per month and moderate to complete improvement in brain fog after starting eptinezumab.

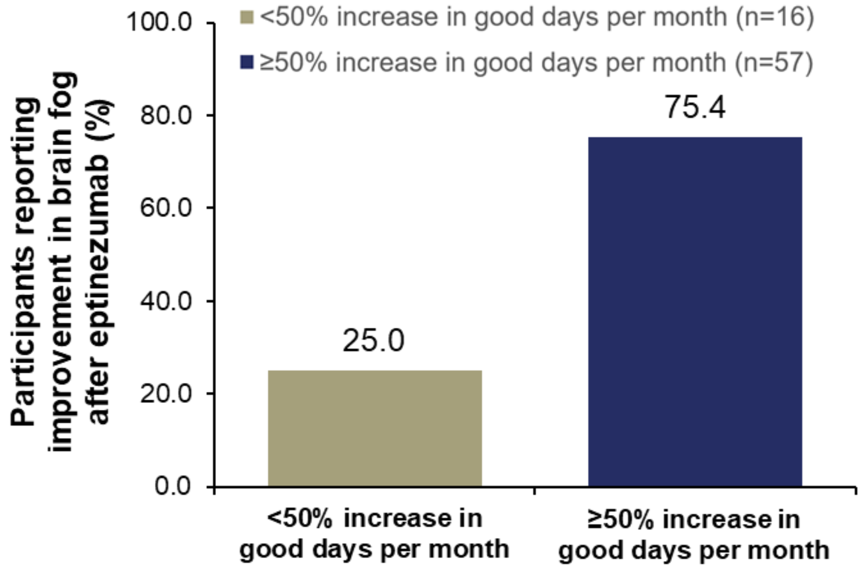

Participant prompt: "Have you experienced 'brain fog' (feeling confused, have difficulty learning or remembering, or have trouble speaking or reading)? If yes, please rate to what extent your symptoms have improved since starting eptinezumab." Moderate to complete improvement included the choices "completely," "very much," and "moderately."
